# FTY720 Prevents Spatial Memory Impairment in a Rat Model of Chronic Cerebral Hypoperfusion via a SIRT3-Independent Pathway

**DOI:** 10.3389/fnagi.2020.593364

**Published:** 2021-01-14

**Authors:** Miao Zhang, Yuan Hu, Jiahui Zhang, Junjian Zhang

**Affiliations:** Department of Neurology, Zhongnan Hospital of Wuhan University, Wuhan, China

**Keywords:** chronic cerebral hypoperfusion (CCH), neuroinflammation, mitochondrial dysfunction, mitophagy, fingolimod

## Abstract

Vascular dementia (VD) and Alzheimer's disease (AD) are the most prevalent types of late-life dementia. Chronic cerebral hypoperfusion (CCH) contributes to both AD and VD. Recently, accumulating evidence has indicated that fingolimod (FTY720) is neuroprotective in acute cerebral ischemic stroke animal models, and the drug is now being used in clinical translation studies. However, fewer studies have addressed the role of FTY720 in chronic cerebral hypoperfusion (CCH)-related brain damage. In the present study, to investigate whether FTY720 can improve CCH-induced spatial memory loss and its underlying mechanism, two-vessel occlusion (2VO) rats were administered intraperitoneal FTY720 (1 mg/kg) for 7 consecutive weeks from post-operative day 8. Spatial memory was tested using the Morris Water Maze (MWM), and the rats' brains were harvested to allow molecular, biochemical, and pathological tests. We found that FTY720 treatment significantly reduced the escape latency and increased the target quadrant swimming time of the 2VO rats in the MWM task. The improvement in memory performance paralleled lower levels of pro-inflammatory cytokines and Iba-1 positive cells in the hippocampus of the 2VO rats, indicating that FTY720 had a beneficial effect in mitigating neuroinflammation. Furthermore, we found that FTY720 alleviated mitochondrial dysfunction in 2VO rats, as manifested by lower malondialdehyde levels, higher ATP content, and upregulation of ATP synthase activity in the hippocampus after treatment. FTY720 had no effect on the CCH-induced decrease in the activity of hippocampal Sirtuin-3, a master regulator of mitochondrial function and neuroinflammation. In summary, the results showed that FTY720 can improve CCH-induced spatial memory loss. The mechanism may involve Sirtuin-3-independent regulation of mitochondrial dysfunction and neuroinflammation in the hippocampus. The present study provides new clues to the pathological mechanism of CCH-induced cognitive impairment.

## Introduction

Chronic cerebral hypoperfusion (CCH) is an important pathophysiological process underlying Alzheimer's disease (AD) and vascular dementia (VD) (Duncombe et al., 2017). Animal models of CCH, including the two-vessel occlusion (2VO) rat model and the bilateral carotid artery stenosis (BCAS) mouse model, mimic the cognitive impairment of AD and VD patients (Venkat et al., 2015; Tuo et al., 2020; Yao et al., 2020). Mitochondrial dysfunction under CCH conditions can induce oxidative stress, as well as neural and synaptic damage, thus triggering microglial activation and astrogliosis (Du et al., 2017). Therefore, mitochondrial dysfunction is a key upstream event for other pathological changes in CCH. Despite this, the mitochondrial mechanism of CCH-induced cognitive impairment remains elusive.

Deacetylase sirtuin-3 (SIRT3) is a master regulator of mitochondrial function (Hirschey et al., 2010). By deacetylating and enhancing the activity of superoxide dismutase-2 (SOD2), isocitrate dehydrogenase-2 (IDH2), and multiple enzymes in the electron transmission chain, SIRT3 can prevent oxidative damage and promote mitochondrial bioenergetics (Yu et al., 2012; Gao et al., 2018). Chronic administration of SIRT3 agonist honokiol (HNK) prevents oxidative stress, neuroinflammation, and spatial memory impairment in the 2VO rat model (Guo et al., 2019). Thus, SIRT3 may be involved in CCH-related mitochondrial dysfunction.

Neurons recycle damaged mitochondria through mitophagy, thus maintaining cellular homeostasis and inhibiting mitochondria-dependent cell apoptosis (Lou et al., 2020). AD manifests mitophagy deficiency, resulting in impaired mitochondrial accumulation, neuronal apoptosis, and ultimately cognitive impairment (Fang et al., 2019). One interesting question is whether CCH can induce a similar pathological change, since it is reported that SIRT3 can induce mitophagy in mouse heart (Li et al., 2018).

Fingolimod (FTY720) was approved as the first oral drug for treating relapsing forms of multiple sclerosis in 2010 (Volpi et al., 2019). It subsequently proved to be a promising treatment for stroke (Wang et al., 2020). The drug binds to sphingosine 1-phosphate receptors (S1PRs) and can exert neuroprotection in multiple models of neurological disease. It can prevent memory loss in rodent models of AD (McManus et al., 2017) and autism (Wu et al., 2017), and it alleviates hypoxic-ischemia injury in the neonatal rat brain (Serdar et al., 2016). This suggests that FTY720 exerts its neuroprotective effects by mitigating oxidative stress and neuroinflammation, which also underlie CCH-related neuronal damage. One recent study further reported that the drug can prevent working memory deficits in a mouse model of BCAS by regulating microglia polarization in the corpus callosum (Qin et al., 2017). However, since BCAS mice show normal spatial memory function, it remains unclear whether FTY720 can prevent CCH-induced hippocampus-dependent spatial memory loss.

We sought to determine whether FTY720 administration during the chronic phase could prevent spatial impairment in the 2VO rat model and, if so, whether SIRT3 or other mitochondrial mechanisms are involved.

## Materials and Methods

### Animals

**Figure 1:**
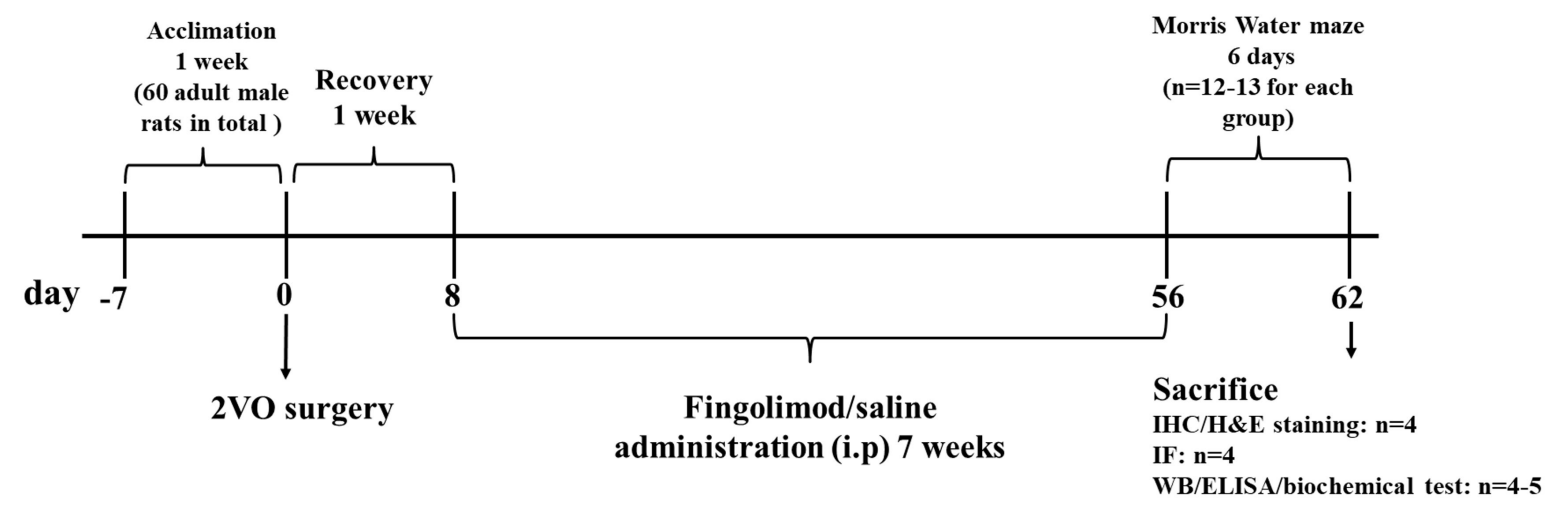
Schematic representation of the experimental design. 2VO, two-vessel occlusion; IHC, immunohistochemistry; H&E staining, hematoxylin and eosin staining; IF, immunofluorescence; WB, western blot; ELISA, Enzyme-linked immunosorbent assay.

Sixty six-week-old male Sprague–Dawley rats weighing 200 ± 20 g were raised in the Animal Experiment Center of Zhongnan Hospital, Wuhan University. They were kept at a temperature of 22 ± 1°C and in a controlled 12-h light/dark cycle. Food and water were available *ad libitum* throughout the study. The rats were randomly divided into four groups after 7 days of acclimation: sham with normal saline administration (sham+NS), sham with FTY720 treatment (sham+FTY720), 2VO with normal saline administration (2VO+NS), and 2VO with FTY720 treatment (2VO+FTY720). The timeframe of this study is presented in Figure 1. All experimental procedures were conducted with permission from the Ethics Committee of Animal Experimentation of Wuhan University and in strict accordance with the ARRIVE Guidelines (Kilkenny et al., 2010).

### Two-Vessel Occlusion Surgery and Drug Administration

The 2VO procedure was performed as previously described (Farkas et al., 2004; Sanderson and Wider, 2013; Hu et al., 2019). After anesthesia induction by inhalation of 4% isoflurane for 3–5 min, rats were maintained under anesthesia using 2% isoflurane (0.5 L/min). A 2-cm incision was made in the middle of the cervical region, with the rat in a supine position. Next, the muscle and fascia were dissected bluntly. The carotid artery was separated and exposed within the carotid sheath. A 4–0 silk suture was used to permanently ligate the carotid. After 30 min, the other carotid was ligated in the same way. Sham groups were subjected to the same procedure without carotid ligation. Rats were placed in a sterilized blanket during the surgery to keep their bodies warm. After skin suturing, the rats were put back into their cages with free access to food and water. FTY720 (F126599; Aladdin) was dissolved in 0.9% normal saline to prepare 10% solutions. After 8 days of surgery, the rats received FTY720 (1 mg/kg) or normal saline treatment by intraperitoneal injection once a day for 7 consecutive weeks. After that, the Morris water maze (MWM) test was carried out.

### Morris Water Maze

The MWM was performed as previously described (Vorhees and Williams, 2006). The round swimming arena was divided into four quadrants with identical areas. A platform was placed 1 cm below the water in the middle of the target quadrant. The rats were gently placed in the water, and the hidden platform was the only escape. The time each rat spent finding the platform was recorded as the escape latency, and swimming speed was also recorded to evaluate locomotive ability. The escape latency of rats that failed to find the platform within 1 min was recorded as 60 s. Whether or not the platform was found, each rat was allowed to stand on the platform for 15 s after the test. During the 5 days of the training phase, the rats were released to swim from four different locations on opposite sides of the platform in sequence. On the 6th day for the probe trial, the platform was removed and rats started from the opposite position where the platform was located. The time spent in the target quadrant was recorded.

### Western Blot

Rats were sacrificed for immunoblotting after the behavioral test. The brain was collected on ice to prepare for hippocampal dissection. Tissues of the hippocampus were immediately frozen in liquid nitrogen and stored at −80°C. RIPA (P0013B; Beyotime Biotechnology, Shanghai, China) buffer with PMSF (ST505; Beyotime Biotechnology, Shanghai, China) was used to lyse the tissues. The homogenate was centrifuged at 10,000 g for 10 min at 4°C. The supernatant was transferred into a new EP tube and prepared for protein concentration using a BCA Protein Assay Kit (P0012S; Beyotime Biotechnology, Shanghai, China). Extracted proteins (20 μg) were separated using 10% sodium dodecyl sulfate polyacrylamide gel electrophoresis and transferred onto a 0.45-μm pore size polyvinylidene fluoride membrane (IPVH00010; Millipore, MA, USA). Next, 5% (weight/volume) skim milk power was added to TBS/0.1% Tween-20 (0.1% TBST) to block non-specific protein-binding on the membrane. Primary antibody was diluted in 0.1% TBST and incubated with the membrane overnight at 4°C. Horseradish peroxidase-conjugated secondary antibody (1:10,000; SA00001-2; Proteintech, Wuhan, China) diluted in 0.1% TBST was used to incubate the membranes at room temperature for 1 h. An enhanced chemiluminescence system (Tanon-5200, Shanghai, China) was used for visualization and semi-quantitation of target protein expression. The following antibodies were used in our research: anti-postsynaptic density-95 (anti-PSD95; 1:1,000; ab18258; Abcam, Cambridge, UK), anti-SIRT3 (1:1,000; 2627s; Cell Signaling Technology, MA, USA), anti-SOD2 (1:1,000; ab68155; Abcam, Cambridge, UK), anti-IKBα (1:2,000, ab32518 Abcam, Cambridge, UK), anti-SOD2 acetyl K68 (1:1,000; ab137037; Abcam, Cambridge, UK), anti-p62 (1:10,000; ab56416; Abcam, Cambridge, UK), anti-cyclooxygenase-4 (COX4; 1:1,000; PA5-29992, Thermo, MA, USA), and anti-GAPDH (1:10,000; Proteintech, Wuhan, China).

### Immunohistochemistry

Immunohistochemistry was performed as previously described (Hu et al., 2019). Anesthetized rats were perfused from the heart with 150 mL 0.9% saline and then 150 mL 4% paraformaldehyde. Brains were dissected, dehydrated, and embedded in paraffin. Paraffin-embedded brains were sectioned coronally into 10-μm thick slices. Antigen retrieval was performed by heating the sections in citrate buffer after deparaffinization and rehydration. Slices were then blocked with 10% goat serum TBS and incubated with primary antibody-ionized calcium-binding adapter molecule 1 (anti-Iba-1; 1:400; 10904-1-AP, Proteintech) overnight at 4°C. Biotinylated secondary antibody was applied for 30 min at room temperature. After that, avidin–biotin complex followed by 3,3′-diaminobenzidine solution (P0202; Beyotime Technology) were applied to the tissues for 30 and 10 min, respectively. Finally, the slices were counterstained using hematoxylin for nuclear staining.

### Hematoxylin and Eosin Staining and Cell Counting

Slices for cell numbers in the cornu ammonis 1 (CA1) region were subjected to hematoxylin and eosin (H&E) staining. The hematoxylin stained the nuclear chromatin, while the eosin stained the cytoplasm. All samples were observed under an Olympus BX53 microscope at 400x magnification. The same CA1 region of the images was captured from three slices in each animal using image analysis software (Olympus Stream). The number of cells and Iba-1-positive cells per mm^2^ in the CA1 region and dentate gyrus (DG) region were calculated using ImageJ software (NIH, USA).

### Immunofluorescence

Immunofluorescence was performed as previously described (Kaiser and Feng, 2019). Anesthetized rats were perfused from the heart with 0.9% saline (150 mL) and then 4% paraformaldehyde. Brains were dissected and submerged in 4% paraformaldehyde overnight at 4°C followed by 30% sucrose/PBS dehydration. The brains were then covered in optimal cutting temperature compound and stored at −80°C to await sectioning into 10 μm-thick slices. Subsequently, the slices were blocked using 10% goat serum TBS and incubated with primary antibodies overnight at 4°C. For mitophagy event detection, fluorescent secondary antibodies from different hosts were applied for 30 min at room temperature. DAPI was applied to mark the nuclei after the secondary antibody was washed off. The primary antibodies used were as follows: anti-LC3 (1:200; ab48394, Abcam), anti-translocase of the outer mitochondrial membrane member-20 (anti-TOMM20; 1:100; ab56783, Abcam). Alexa Fluor 594-conjugated secondary antibody (1:200; ab150080; Abcam) and Alexa Fluor 488-conjugated (cross-adsorbed) secondary antibody (1:100; A11029, Invitrogen) were applied. Anti-PSD95 (1:200; ab18258; Abcam, Cambridge, UK) primary antibody and Alexa Fluor 594-conjugated secondary antibody (1:200; ab150080; Abcam) were applied for synaptic staining. All samples were observed under an Olympus BX53 microscope at 400x magnification. The same CA1 region of the images was captured from three slices in each animal using image analysis software (Olympus Stream). Area Fraction of PSD95 and colocation of LC3 and TOMM20 was identified using Image J software (NIH, USA).

### Enzyme-Linked Immunosorbent Assay

Levels of tumor necrosis factor (TNF-α), interleukin-1β (IL-1β), and interleukin-6 (IL-6) in the rat hippocampus were analyzed using an enzyme-linked immunosorbent assay (ELISA) kit according to the manufacturer's instructions, with the optical density value measured using a microplate reader (DR-200Bs; DiaTek Company, Wuxi, China) at a wavelength of 450 nm. The following ELISA kits were used: Rat TNF-α ELISA Kit (ELK1396; ELK Biotechnology, Wuhan, China), Rat IL-1β ELISA Kit (ELK1272 ELK Biotechnology, Wuhan, China), and Rat IL-6 ELISA Kit (ELK5684; ELK Biotechnology, Wuhan, China).

### Biochemical Detection

The levels of ATP, SOD2, and ATP synthase activity in the hippocampal homogenate of rats were detected using colorimetric assay kits (Nanjing Jiancheng Bioengineering Research Institute, Nanjing, China), according to the manufacturer's instructions. A spectrophotometer was used in the test process. The following assay kits were used: SOD2 activity (A001-2), ATP content (A095-1-1), Mitochondrial extraction (G006-1-1), and ATP synthase activity (A089-5-1).

### Statistical Analysis

Data are presented as mean ± standard error of the mean. The Shapiro-Wilk test did not show a significant departure from normality in the distribution of the parameters. Statistical analyses were performed using GraphPad Prism 6 software (GraphPad Software, USA). In western blot, immunohistochemistry, ELISA, biochemical analysis, and part of the MWM test (time spent in target quadrant of probe trial; swimming speed), differences between groups were determined using one-way analysis of variance (ANOVA) followed by the *post-hoc* Bonferroni test. Differences in escape latency during the five training periods were analyzed using two-way ANOVA, and *p* < 0.05 were considered statistically significant.

## Results

### FTY720 Attenuated Spatial Memory Impairment Induced by CCH in 2VO Rats

**Figure 2:**
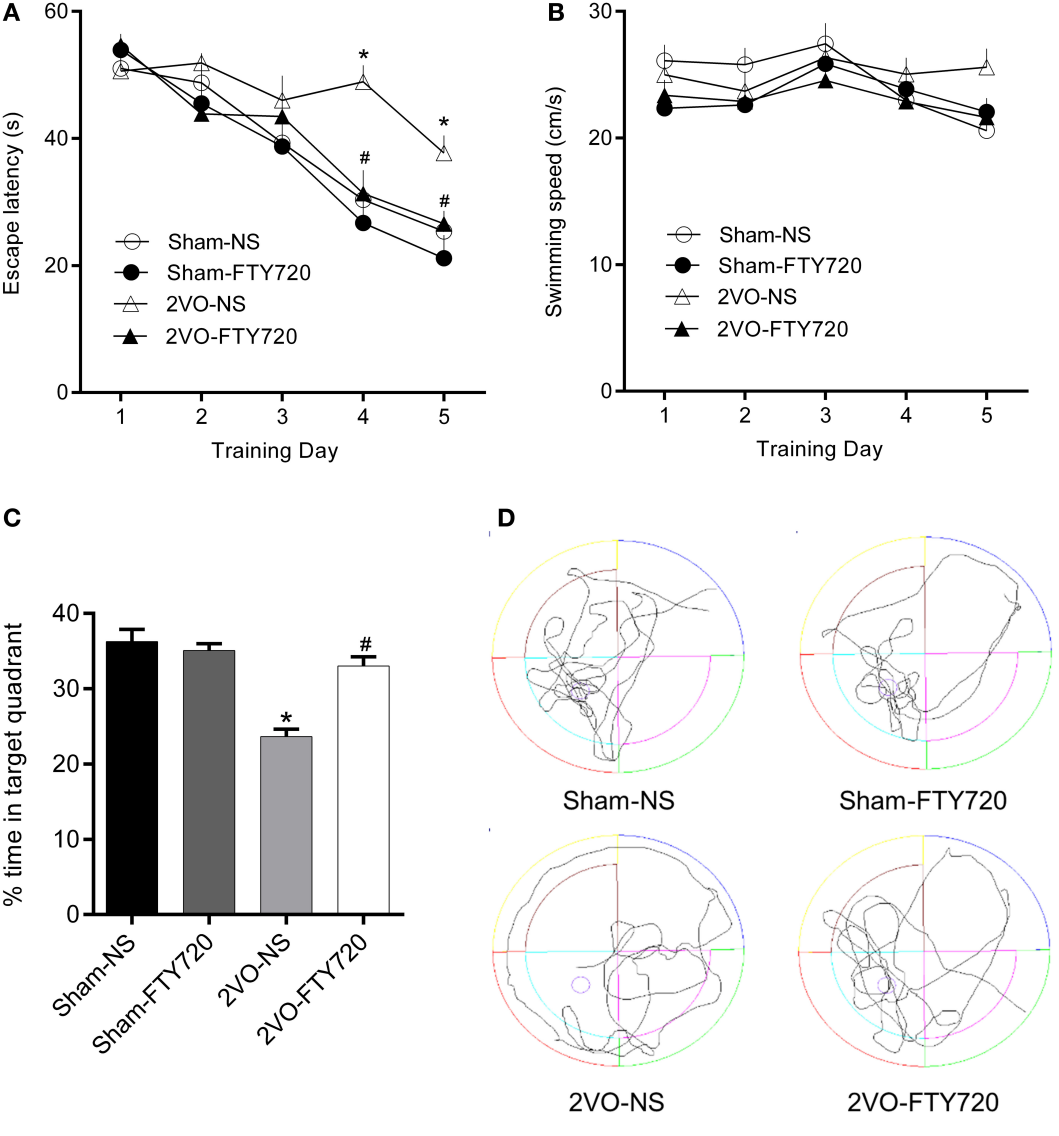
The escape latency **(A)**, swimming speed during the training phase, **(B)** percentage of time spent in target quadrant **(C)**, and representative swimming paths **(D)** in the probe trial of MWM task of FTY720 or Saline treated rats after the 2VO surgery. All data are shown as mean ± S.E.M, *n* = 12–13 rats for each group. *compared to sham-NS rats, *p* < 0.05, ^#^compared to 2VO-NS rats, *p* < 0.05.

The MWM was applied to test the spatial memory of rats in the four groups. Figure 2A shows that, over the 5 consecutive days of the training phase, there was no significant difference in escape latency in the first 3 days, while 2VO-NS rats spent more time finding the platform than the 2VO-FTY720 rats on the 4th and 5th days (*p* < 0.05). On the probe trial phase, 2VO-FTY720 rats exhibited a higher percentage of time in the target quadrant than 2VO-NS rats (*p* < 0.05; Figure 2C). There was no significant difference in swimming speed among the four groups (*p* > 0.05; Figure 2B). Representative swimming paths were shown in Figure 2D. These results suggested that FTY720 improved spatial memory in 2VO rats without affecting their locomotive ability.

### FTY720 Did Not Affect Hippocampus CA1 Neuron Loss in 2VO Rats

**Figure 3:**
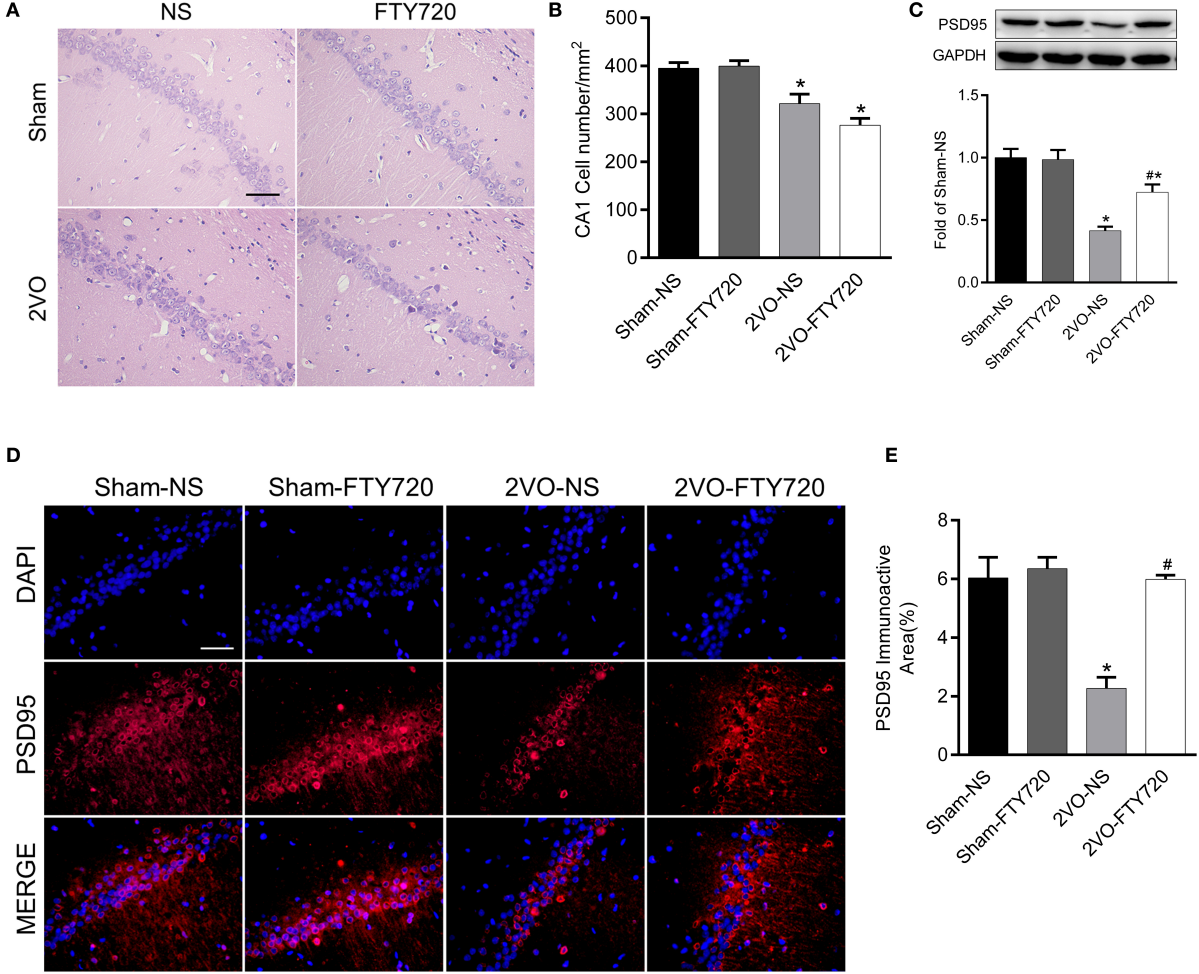
The effect of FTY720 on CA1 cell number and PSD95 expression. **(A)** Representative H&E staining image of rat hippocampus CA1 region (Scale bar = 50 μm, 400×). **(B)** Statistical bar graph of cell numbers in hippocampus CA1 region. **(C)** Representative western blot bands of rat hippocampal PSD95 and quantitative analysis. Data was normalized to GAPDH and expressed as fold of sham-NS **(D,E)** Representative image and statistical analysis of PSD95 immunofluorescence staining (Scale bar = 50 μm, 400×). Data were shown as mean ± S.E.M. *n* = 4 rats for each group. *p < 0.05 compared with the sham-NS group. ^#^*p* < 0.05 compared with the 2VO-NS group.

H&E staining of the hippocampus CA1 region showed that the cell number was lower in 2VO-NS rats than in sham-NS rats (*p* < 0.05; Figures 3A,B). Compared with 2VO-NS rats, 2VO-FTY720 rats had a tendency toward fewer hippocampal CA1 neurons, although the difference was not significant (*p* > 0.05; Figures 3A,B). To further study whether FTY720 influenced CCH-induced synaptic damage, we tested PSD95 expression in the hippocampus by western blotting. As shown in Figure 3C, PSD95 expression was lower in 2VO-NS rats than in sham-NS rats (*p* < 0.05), while FTY720 administration conferred higher PSD95 expression in 2VO rats (*p* < 0.05). Immunofluorescence was conducted to further confirm PSD95 expression. In agreement with the western blot results, FTY720 intervention increased the hippocampal PSD95 immunoactive area in 2VO rats (*p* < 0.05, Figures 3D,E).

### FTY720 Alleviated CCH-Induced Neuroinflammation in the Hippocampus of 2VO Rats

**Figure 4:**
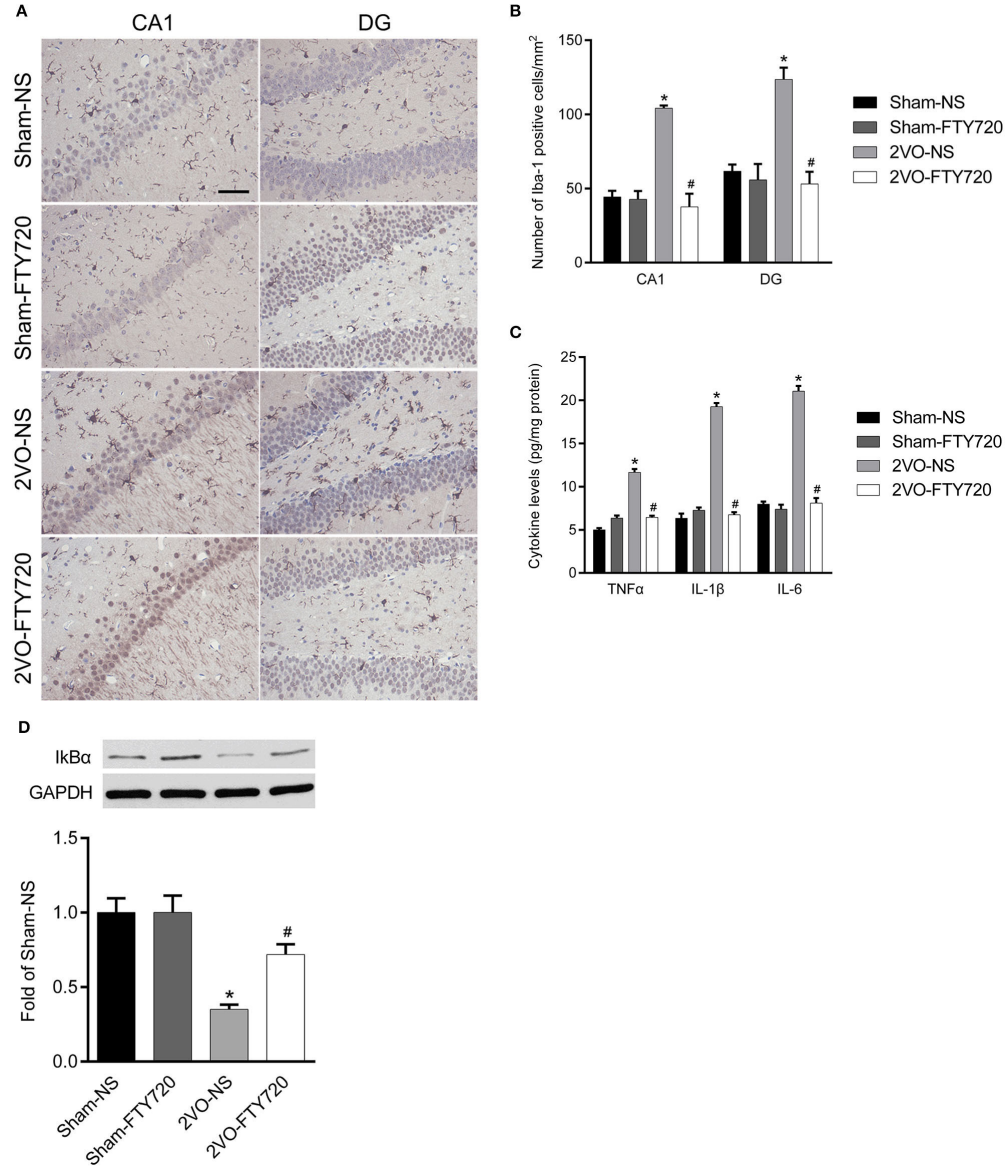
The modulation of FTY720 to CCH-induced hippocampal neuroinflammatory response. **(A)** Representative immunohistochemistry images of Iba-1 positive cells in the hippocampus of rats (Scale bar = 50 μm, 400×). **(B)** Quantitation of Iba-1 positive cells in the hippocampus CA1 and DG regions with bar graph. **(C)** Quantitative bar graph of pro-inflammation cytokines expression in the hippocampus. **(D)** Representative IkBα western blot bands in the hippocampus of animals. Protein level was normalized to the loading of GAPDH and expressed as fold of sham-NS. Data were shown as mean ± S.E.M. *n* = 4–5 rats for each group. *p < 0.05 compared with the sham-NS group. ^#^*p* < 0.05 compared with the 2VO-NS group.

Iba-1 is a marker of microglia, so we counted Iba-1-positive cells in the hippocampal CA1 and DG regions. The 2VO-NS rats expressed more Iba-1-positive cells than the Sham-NS rats in the hippocampus CA1 and DG regions (*p* < 0.05; Figures 4A,B). FTY720 significantly reduced the number of Iba-1-positive cells in both hippocampus subregions in 2VO rats (*p* < 0.05; Figures 4A,B). We then tested the levels of pro-inflammatory cytokines, including TNF-α, IL-6, and IL-1β, using ELISA. The 2VO-NS rats showed higher expression of these pro-inflammatory factors in the hippocampus (*p* < 0.05; Figure 4C), and FTY720 treatment reduced TNF-α, IL-1β, and IL-6 release in the hippocampus of 2VO rats (*p* < 0.05; Figure 4C). Since IKBα inhibits the release of downstream inflammatory cytokines (Oeckinghaus et al., 2011), we also tested the expression of IKBα and found that 2VO-NS rats showed lower IKBα expression in the hippocampus, while FTY720 treatment upregulated IKBα levels (*p* < 0.05; Figure 4D). Overall, these data suggested that FTY720 decreased microglia activation and regulated the concentration of inflammatory factors in response to CCH.

### FTY720 Improved Mitochondrial Dysfunction in the Hippocampus of 2VO Rats

**Figure 5:**
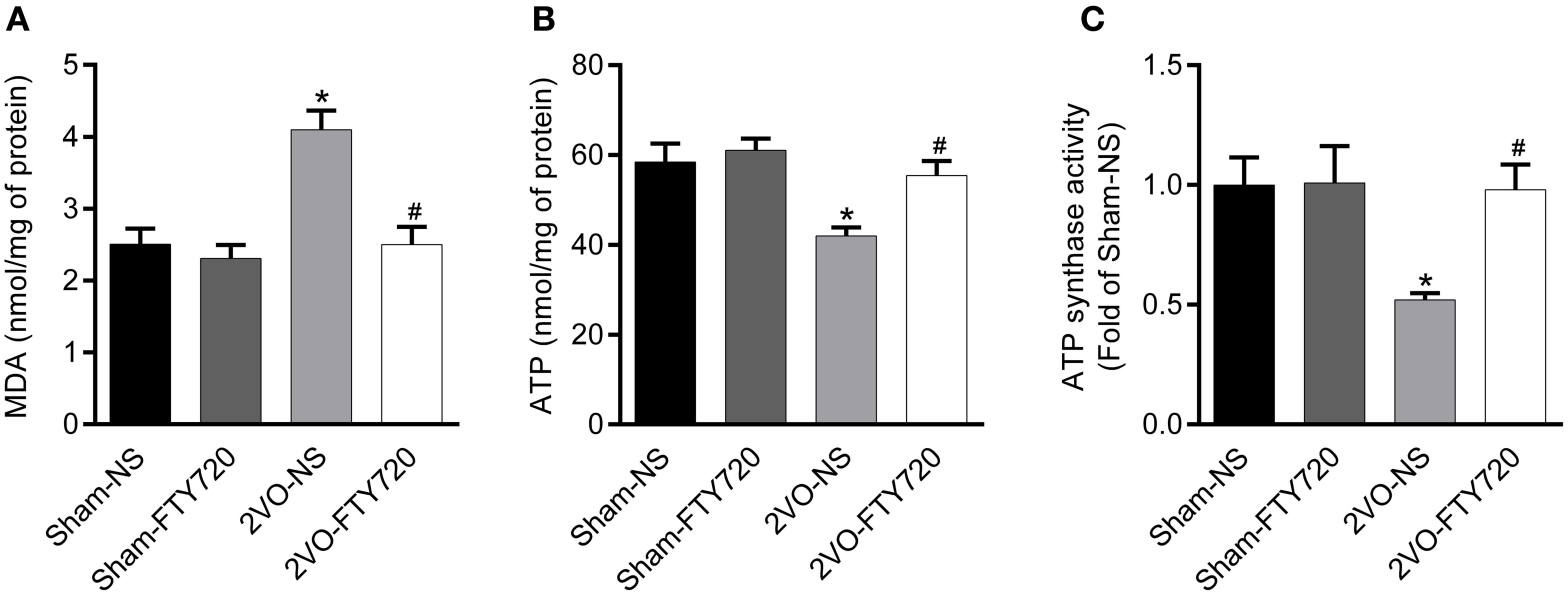
Quantitation of MDA **(A)**, ATP content **(B)** as well as ATP synthase activity **(C)** in the hippocampus of FTY720 or saline treated rats after the 2VO surgery. Data are shown as mean ± S.E.M. *n* = 4–5 rats for each group. *p < 0.05 compared with the sham-NS group. ^#^*p* < 0.05 compared with the 2VO-NS group.

Oxidative stress originating from mitochondrial dysfunction is considered a crucial pathological process in CCH (Du et al., 2013; Ham and Raju, 2017). To further explore whether FTY720 could alleviate hippocampal mitochondrial dysfunction in 2VO rats, we tested malondialdehyde (MDA) and ATP content, as well as ATP synthase activity in the hippocampus of all groups of rats. The 2VO-NS rats showed increased MDA levels (*p* < 0.05; Figure 5A), as well as decreased ATP content and ATP synthase activity compared to Sham-NS and Sham-FTY720 rats (*p* < 0.05; Figures 5B,C). Meanwhile, FTY720 decreased the MDA levels, but increased the ATP content and ATP synthase activity in the 2VO rat hippocampus (*p* < 0.05; Figures 5A–C). These results indicated that FTY720 could ameliorate the hippocampal mitochondrial dysfunction induced by CCH.

### FTY720 Had No Effect on SIRT3 Activity in the Hippocampus of 2VO Rats

**Figure 6:**
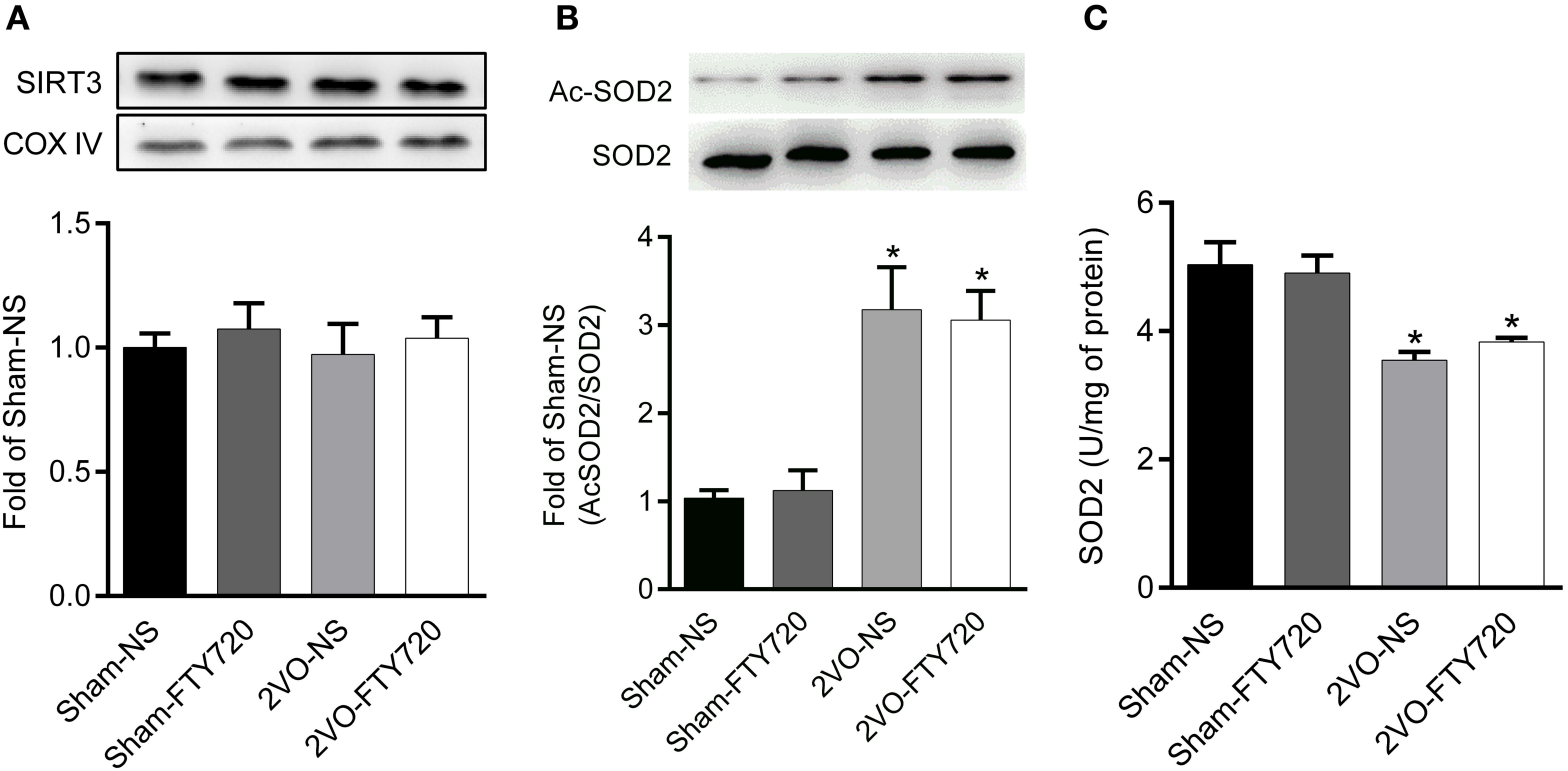
Representative SIRT3 **(A)**, acetylated SOD2 K68 (Ac-SOD2 K68) **(B)** expressions, SOD2 activity **(C)** in the hippocampus of rats in each group. SIRT3 and SOD2 K68 expression in the hippocampus were normalized to loading of COX IV and SOD2, respectively, and were expressed as fold of sham-NS. Data were shown as mean ± S.E.M. *n* = 4–5 rats for each group. *p < 0.05 compared with the sham-NS group.

There was no significant difference in SIRT3 protein expression among the four groups after FTY720 treatment (*p* > 0.05; Figure 6A). SOD2 is the downstream target molecule of SIRT3. We found that 2VO-NS and 2VO-FTY720 rats showed higher acetylated-SOD2 K68 levels than controls in the hippocampus (*p* < 0.05; Figure 6B). However, FTY720 treatment did not improve acetylated-SOD2 K68 levels in 2VO rats (*p* < 0.05; Figure 6B). Consistent with the SOD2 acetylation level, FTY720 did not increase SOD2 activity in the hippocampus of 2VO rats (Figure 6C).

### FTY720 Did Not Influence Mitophagy in the Hippocampus CA1 Region of 2VO Rats

**Figure 7:**
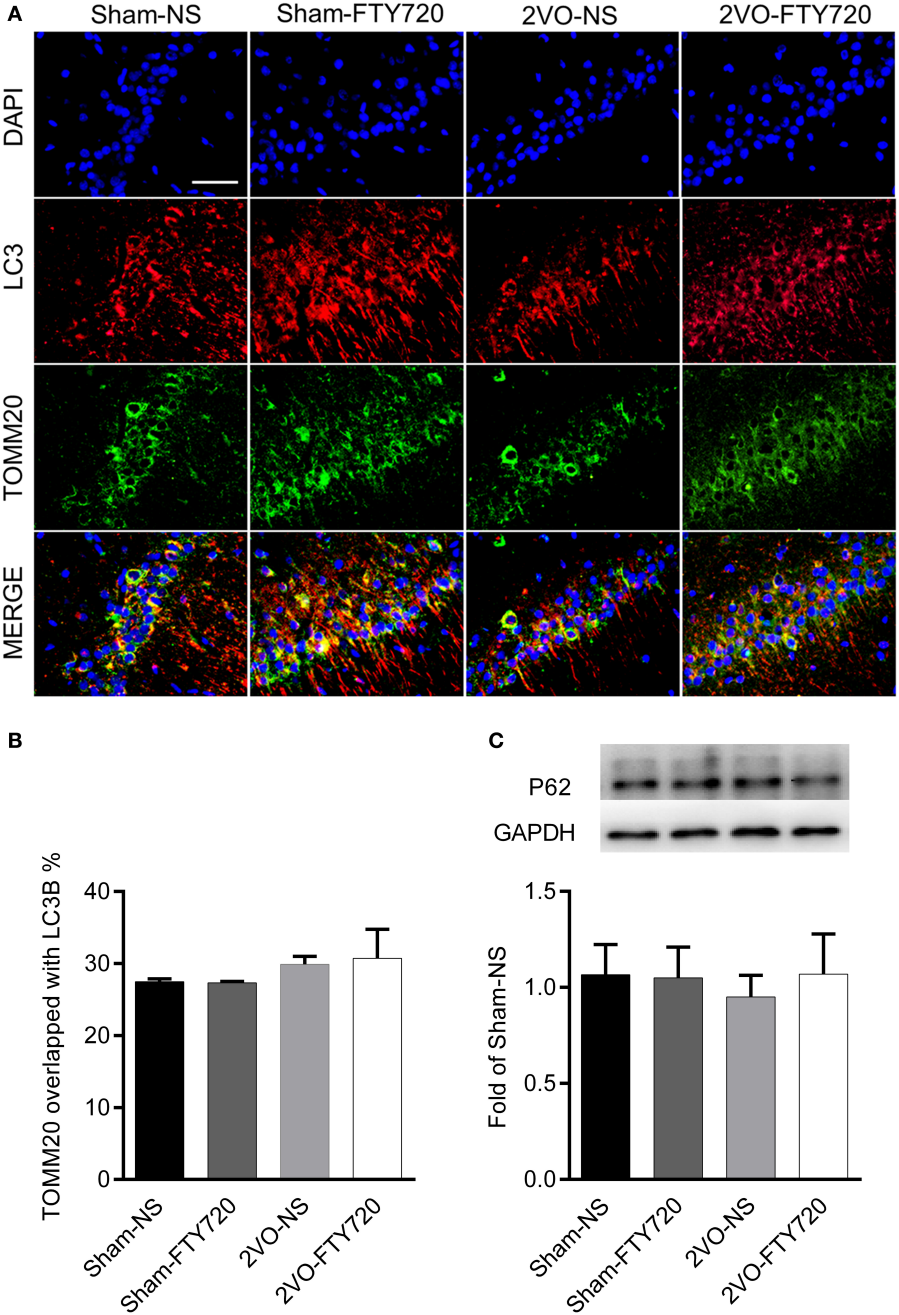
The effect of FTY720 on hippocampal mitophagy markers of 2VO rats. **(A)** Representative immunofluorescent images of LC3 and TOMM20 colocalization in the hippocampus CA1 region. **(B)** The effect of FTY720 on hippocampal mitophagy markers of 2VO rats. **(C)** Representative hippocampal p62 immunoblot bands of FTY720 or saline treated rats after the 2VO surgery. p62 levels were normalized to GAPDH and expressed as fold of sham-NS in the lower panel. All data were shown as mean ± S.E.M. *n* = 4–5 rats for each group. Scale bar=50 μm, 400×.

The autophagy markers LC3 and mitochondria marker TOMM20 were stained in the CA1 region (Figure 7A). TOMM20 overlapping with LC3 ratio was identified as the mitophagy event. However, we found no difference in the overlapping rate of TOMM20 and LC3 among the four groups (*p* > 0.05; Figure 7B), and there was no significant difference among the four groups in the autophagic flux marker p62 (*p* > 0.05; Figure 7C).

## Discussion

The present study proved that FTY720 can prevent CCH-induced spatial memory impairment and mitochondrial dysfunction, without affecting SIRT3 activity.

Bilateral common carotid artery occlusion results in blood flow redistribution in the Willis arterial circle in rats (Farkas et al., 2007). The chronic brain ischemia phase ranges from 4 days to 8–12 weeks after the surgery. The mortality rate reported in previous studies of 2VO surgery has varied from 26 to 50%, and it stops increasing after 1 week (Wang et al., 2014; Li et al., 2015). Therefore, we chose to start drug administration on the 8th day after surgery to avoid any acute ischemia effect.

During the chronic cerebral ischemia phase, neuron death and synapse loss mirror cognitive impairment was induced by CCH (Du et al., 2017). In the present study, FTY720 had no effect on neuron numbers in the hippocampal CA1 region after 2VO, while it has protected against neuronal loss in other studies (Asle-Rousta et al., 2013; Wu et al., 2017). This discrepancy may have arisen because FTY720 intervention was started at different times. Cechetti et al. (2012) calculated neuron numbers in the hippocampal CA1 region at multiple time points, finding that the number of NeuN-positive cells was markedly lower than in sham-operated rats 1 week after surgery. However, there was no significant difference in NeuN-labeled cell number between 1 week and 3 months after surgery (Cechetti et al., 2012), suggesting that no significant neuronal apoptosis occurs during this chronic cerebral ischemia phase. In the present study, we chose to use FTY720 8 days after surgery, so the drug could not prevent early neuron loss. In other neurological disease models in which neuronal loss has been ameliorated by FTY720, preconditioning or early drug administration before neural damage have been applied (Asle-Rousta et al., 2013; Ren et al., 2017).

Neuronal loss normally parallels a decrease in synaptic density, and synaptic function is of vital importance for cognition (Li et al., 2019; Wang et al., 2019; Che et al., 2020). CCH results in a low oxygen supply to the electron transport chain, generating excessive reactive oxygen species (ROS), inducing oxidative stress, and thus facilitating neuroinflammation in the hippocampus (Farkas et al., 2007). According to previous studies (Rao et al., 2012; Bollinger et al., 2019), oxidative stress and neuroinflammation cause synaptic damage. FTY720 has been reported to mitigate oxidative stress and neuroinflammatory reactions in animal models of neurological diseases (Colombo et al., 2014; Serdar et al., 2016). Corroborating this, our results showed that FTY720 alleviated oxidative stress and neuroinflammation in the hippocampal CA1 region of 2VO rats, and that it increased PSD95 and recovery of memory deficits. It follows that mitigated oxidative stress and neuroinflammation, as well as subsequent improved synaptic function, underlies the cognitive protective effect of FTY720.

Mitophagy is a key process to guarantee normal mitochondrial function by alleviating oxidative stress (Baechler et al., 2019; Zhang et al., 2019). Recently, mitochondrial damage due to mitophagy reduction has been revealed as an important factor in AD-related cognitive impairment (Fang et al., 2019). In the present study, there was no change in colocalization of the autophagy marker LC3 and the mitochondrial membrane protein TOMM20 after FTY720 administration, indicating that FTY720 has no impact on mitophagy. Expression of p62 remained unchanged after FTY720 intervention, indicating that FTY720 does not affect autophagic flux, since p62 is important in the transfer of ubiquitinated substrates to autophagosomes (Wen et al., 2019). Therefore, mitophagy may not be the main mechanism responsible for mitochondrial dysfunction in CCH, as it is in AD.

The mitochondrial deacetylase SIRT3 is the master regulator of mitochondrial function (Baeza et al., 2016; Salvatori et al., 2017; Gao et al., 2018). SIRT3 deficiency aggravates cerebral ischemia-induced oxidative stress and neuroinflammation (Yang et al., 2020). Chronic administration of SIRT3 agonist honokiol (HNK) also prevents oxidative stress, neuroinflammation, and spatial memory impairment in 2VO rats (Guo et al., 2019). As the downstream molecule, SOD2 is acetylated by SIRT3, so its K68 acetylation level can reflect SIRT3 activity (Chen et al., 2011). Our research found that SOD2 K68 acetylation level, rather than SIRT3 protein level, decreased in the CCH rat hippocampus. This result indicates that SIRT3 post-translational change may be involved in CCH-induced mitochondrial dysfunction. Since SIRT3 can be post-translationally modified through phosphorylation or S-sulfhydration (Liu et al., 2015; Yuan et al., 2019), further study is needed to ascertain how CCH-induced post-translational modification of SIRT3 is related to cognitive impairment. FTY720 does not influence SIRT3 protein expression or its activity in the hippocampus of 2VO rats, indicating that chronic FTY720 administration involves an alternative pathway.

One limitation of the present study was the lack of further study into the mechanism by which FTY720 ameliorates CCH-induced mitochondrial dysfunction. A recent *in vitro* study found that FTY720 can increase neuronal mitochondrial stability and restore mitochondrial dynamics under conditions of oxidative stress by modulating S1PRs (Martin-Montanez et al., 2019). Further studies are needed to clarify how membrane-anchored S1PRs can couple with mitochondria when *in vivo* specific S1PR agonists/antagonists are available.

In conclusion, the present study demonstrated that FTY720 can prevent CCH-induced spatial memory impairment, oxidative stress, and neuroinflammation, possibly by activating SIRT3-independent mitochondrial pathway.

## Data Availability Statement

The datasets analyzed in this article are not publicly available. Requests to access the datasets should be directed to Miao Zhang, milkyzm@163.com.

## Ethics Statement

The animal study was reviewed and approved by Ethics Committee of Animal Experimentation of Wuhan University.

## Author Contributions

MZ and YH conceived the study and performed the experiments. JiZ analyzed the data. JuZ drafted the manuscript. All authors edited and approved the manuscript.

## Conflict of Interest

The authors declare that the research was conducted in the absence of any commercial or financial relationships that could be construed as a potential conflict of interest.
